# Meta-analysis of genes within QTLs of group A streptococcal sepsis and their expression QTLs reveal pathways modulating host differential response to streptococcal sepsis

**DOI:** 10.1186/1471-2105-13-S12-A6

**Published:** 2012-07-31

**Authors:** Nourtan Abdeltawab, Lu Lu, Robert Williams, Malak Kotb

**Affiliations:** 1 Veteran’s Affairs Medical Center, Cincinnati, OH 45220, USA; 2 Department of Anatomy and Neurobiology, University of Tennessee Health Science Center, Memphis, TN 38163, USA; 3 Faculty of Pharmacy, Cairo University, Cairo, Egypt; 4 Department of Molecular Genetics, Microbiology and Immunology, University of Cincinnati College of Medicine, Cincinnati, OH 45267, USA

## 

Complex host–pathogen interactions modulate differential responses to group A streptococcal (GAS) sepsis systemic disease [1,2]. We previously found that host HLA-II allelic variations are associated with differential response to severe GAS sepsis [3]. In addition, using mouse models of GAS sepsis we found other host genetic factors contribute to disease severity by modulating inflammatory responses [4,5]. We applied systems genetics approaches and analyzed variations in disease severity phenotypes using advance recombinant inbred (ARI) BXD strain panel. We mapped quantitative trait loci (QTLs) associated with differential host response to severe GAS sepsis to mouse Chr2 and X [5]. The focus of the current study is to identify regulating genes within QTLs associated with differential GAS sepsis. To do so, we explored differences in expression and nsSNPs of genes within mapped QTLs using expression data sets of relevant tissues. We selected spleen, leukocytes and lung expression data sets deposited in GeneNetwork as most relevant data sets for GAS sepsis disease severity. Collectively, integration of QTL mapping of sepsis phenotypes with expression QTLs uncovered pathways that modulate differential susceptibility to severe GAS sepsis, underscoring the complexity of traits modulating severe GAS sepsis. Approaches used in our study provide a powerful, unbiased genetics approach for analyzing interactive traits modulating the outcomes of infectious diseases.

## References

[B1] Cunningham MW Pathogenesis of group A streptococcal infections and their sequelae Adv Exp Med Biol 2008 609 29 42 10.1007/978-0-387-73960-1_3 18193655

[B2] Cunningham MW Pathogenesis of group A streptococcal infections Clin Microbiol Rev 2000 13 3 470 511 10.1128/CMR.13.3.470-511.2000 10885988 PMC88944

[B3] Kotb M Norrby-Teglund A McGeer A El-Sherbini H Dorak MT Khurshid A Green K Peeples J Wade J Thomson G An immunogenetic and molecular basis for differences in outcomes of invasive group A streptococcal infections Nat Med 2002 8 12 1398 1404 10.1038/nm1202-800 12436116

[B4] Nooh MM El-Gengehi N Kansal R David CS Kotb M HLA transgenic mice provide evidence for a direct and dominant role of HLA class II variation in modulating the severity of streptococcal sepsis J Immunol 2007 178 5 3076 3083 17312154 10.4049/jimmunol.178.5.3076

[B5] Abdeltawab NF Aziz RK Kansal R Rowe SL Su Y Gardner L Brannen C Nooh MM Attia RR Abdelsamed HA An unbiased systems genetics approach to mapping genetic loci modulating susceptibility to severe streptococcal sepsis PLoS Pathog 2008 4 4 e1000042 10.1371/journal.ppat.1000042 18421376 PMC2277464

